# Maintenance care in chiropractic – what do we know?

**DOI:** 10.1186/1746-1340-16-3

**Published:** 2008-05-08

**Authors:** Charlotte Leboeuf-Yde, Lise Hestbæk

**Affiliations:** 1 Nordic Institute of Chiropractic and Clinical Biomechanics, part of Clinical Locomotion Science, University of Southern Denmark, Forskerparken 10, DK-5230 Odense M, Denmark

## Abstract

**Background:**

Back problems are often recurring or chronic. It is therefore not surprising that chiropractors wish to prevent their return or reduce their impact. This is often attempted with a long-term treatment strategy, commonly called maintenance care. However, some aspects of maintenance care are considered controversial. It is therefore relevant to investigate the scientific evidence forming the basis for its use.

**Objectives:**

A review of the literature was performed in order to obtain answers to the following questions: What is the exact definition of maintenance care, what are its indications for use, and how is it practised? How common is it that chiropractors support the concept of maintenance care, and how well accepted is it by patients? How frequently is maintenance care used, and what factors are associated with its use? Is maintenance care a clinically valid method of approach, and is it cost-effective for the patient?

**Results:**

Thirteen original studies were found, in which maintenance care was investigated. The relative paucity of studies, the obvious bias in many of these, the lack of exhaustive information, and the diversity of findings made it impossible to answer any of the questions.

**Conclusion:**

There is no evidence-based definition of maintenance care and the indications for and nature of its use remains to be clearly stated. It is likely that many chiropractors believe in the usefulness of maintenance care but it seems to be less well accepted by their patients. The prevalence with which maintenance care is used has not been established. Efficacy and cost-effectiveness of maintenance care for various types of conditions are unknown. Therefore, our conclusion is identical to that of a similar review published in 1996, namely that maintenance care is not well researched and that it needs to be investigated from several angles before the method is subjected to a multi-centre trial.

## Background

Chiropractors all over the world are consulted for spinal pain and dysfunction. Because many spinal pain complaints are chronic or recurrent in nature [1,2], it is understandable that, once improvement has been achieved, chiropractors attempt to prevent new events or maintain patients at their optimal level. This is usually done by scheduling additional visits over a prolonged period of time but at longer time intervals than during the acute event. Among chiropractors, this approach is named "maintenance care", whereas in public health terms it is described as secondary or tertiary prevention. Secondary prevention is aimed at preventing new events, whereas tertiary prevention means that improved patients with incurable conditions are maintained at the best possible level.

Although it appears perfectly logical to use maintenance care in chronic and recurrent conditions, when informally discussing this phenomenon with chiropractors, we have often detected either a disinclination to discuss, or an ardour of arguments, often resulting in an embarrassing change of subject. In other words, maintenance care appears to be, for some, a politically incorrect topic.

This might be because the indications for treatment in asymptomatic patients depend solely on tests and observations, such as palpation findings, none of which has been shown to be clearly valid [3]. When treating an acute problem, however, this lack of valid examination tests is of little or no concern, as the patient's reaction to the treatment will provide feedback on the construct validity of the various treatment procedures. Therefore, there appears to be disagreement among chiropractors as to whether chiropractic treatment is mainly effective in the acute phase or whether it is possible also to prevent the underlying disorder, regardless of whether the patient is symptomatic at the time of examination and treatment.

Jamison has discussed the preventive aspect of maintenance care, when encompassing other than the musculoskeletal conditions. She points out that some chiropractors believe "that subluxations can cause, and spinal adjustments correct, diverse problems ranging from pain to more subtle endocrine, visceral and autonomic dysfunctions" and warns that this scientifically untested theory has considerable ill effects in the scientific and medical communities [4]. In general, if chiropractors believe that "spinal health" equals good health, it is understandable that they would try to convince patients to have regular preventive chiropractic treatments. Jamison discusses this in a second paper, where she also mentions the negative repercussions of such practice [5]. It could also be that the overzealous use of maintenance care has resulted in problems with various reimbursement systems, as Mitchell warned already in 1980 [6]. Some individuals' short-term financial gains could be seen as having negative long-term repercussions for the whole profession.

The concept of maintenance care, therefore, seems to be associated with the very core of disagreement between chiropractors and their styles of practice; those who treat mainly musculoskeletal conditions and those who attempt to treat also other conditions. In addition, it may divide those who believe that their examination method is objective and valid and those who depend (also) on patients' signs and symptoms for their diagnosis and treatment.

Nevertheless, maintenance care seems to be commonly employed, and if it is a useful model of preventive treatment, it should be recognized as such; but if it is ineffective, it should not be part of the chiropractic patient management strategy. Maintenance care therefore, merits being taken seriously and to be subjected to scientific scrutiny.

In 1993, the Mercy Guidelines [7] attempted to perform a literature review on this subject but ended up making its recommendations largely on clinical experience "of nearly 100 years". The report suggested that the use of chiropractic adjustments in a regiment of preventive/maintenance care has merit. There are no statements in the guideline in relation to indications, type of treatment, duration and frequency of treatment, nor on effectiveness. It is merely written that maintenance care is "discretionary and elective on the part of the patient" and that when recommended, "it is necessary for the practitioner to clearly identify the type and nature of this care and to give proper patient disclosure".

Aker and Martel, three years later, performed a narrative review and concluded on the basis of the sparse literature that "there is no scientific evidence to support the claim that maintenance care improves health status" and went on to recommend a series of research actions to be taken [8]. Our continued monitoring of the literature revealed several additional studies since the time of their publication.

## Objectives

Therefore, a new literature review of this subject appeared timely, with the intent of:

1. Defining maintenance care and the indications for and nature of its use.

2. Describing to what degree chiropractors believe in maintenance care and to what degree it is accepted by patients.

3. Establishing the prevalence with which chiropractors use maintenance care, and factors associated with its use.

4. Determining its efficacy and cost-effectiveness for various types of conditions.

Because of the few articles that could be traced in relation to the number of questions, only a narrative review could be undertaken.

### Search strategy and inclusion criteria

A librarian-assisted electronic literature search was attempted using the Medline and Mantis databases, with no limitations for language or time period. The search terms were "chiropractic", "maintenance care", and "prevention", both as Mesh-term and free text. However, fewer articles than those already known by the authors appeared, and therefore a manual search was undertaken as well. The index lists of chiropractic journals were examined, as well as reference lists of articles on the topic, which resulted in the 13 articles used in this review. In addition, guidelines and chiropractic textbooks were consulted, but not exhaustively, in order to bring forth some background information on the topic.

The only inclusion criteria for this review were that the texts were research articles published in journals, that they dealt with chiropractors or chiropractic patients, and that they discussed the issue of maintenance care, also described as secondary prevention, tertiary prevention, or regular, long-term chiropractic care. Articles exclusively dealing with primary prevention were not eligible for the review.

## Results

### General description of the studies

We found 13 original articles, in which the issue of maintenance care was investigated. Eleven could be described as surveys and have been briefly described in Table 1 [Additional file 1]. Eight were cross-sectional [4,5,9-14], one was a file inspection study [15], one was a prospective study [16], and one was a one-year multi-centre prospective outcome study, in which the prevalence of maintenance care was retrospectively investigated at the one-year follow-up [17]. The two non-survey articles consisted of a case-report [18] and a preliminary randomized controlled clinical trial, investigating the efficacy of maintenance care in patients with chronic LBP [19]. The first of the studies was published in 1976 with data collected in 1973–4 [15] and the last 30 years later [19].

Five of the surveys were from Australia, three from the USA, two from UK, and one from Norway (Table 1 [Additional file 1]). The clinical trial would have been carried out in Canada [19], whereas it is unclear in which country the case-report was produced [18]. The sample sizes ranged from a case-report of 1 patient [18] to 2056 case files [15].

Five of the 11 surveys had low response rates: 20% [5], 22% [4], 35% [13], 44% [11], 51% [10], and in one survey the response rate does not appear to have been reported [12]. In the file inspection study, the proportion of participants was higher (71%) than the percentage of practitioners whose files were inspected (35%) [15]. Two of the surveys with higher response rates included specific study samples that nevertheless probably did not represent the general chiropractic profession; one of recently graduated Australian chiropractors [9] and one in which Australian chiropractors were included on the basis of whether they practised in a chiropractor-dense area or not [16].

In summary, there were only few studies, covering a wide time-period, unevenly distributed across the world, and the study participants were often unlikely to be representative of their target populations.

### Definitions of maintenance care

The definitions of maintenance care that we found were not based on scientific evidence of the clinical validity of maintenance care but perhaps more on opinion and consensus.

Because there is a conviction among some chiropractors that spinal adjustments/manipulations have a preventive effect not only on musculoskeletal problems but also on the general state of health, it is not surprising that one "officially recognized and approved" definition of maintenance care that we found was rather vague. This would allow the chiropractor to apply it according to his/her own scope of practice. The definition is "Appropriate treatment directed toward maintaining optimal body function. This is treatment of the symptomatic patient who has reached pre-clinical status or maximum medical improvement, where condition is resolved or stable" [20].

Another definition found in the literature is "...a regimen designed to provide for the patient's continued well-being or for maintaining the optimum state of health while minimizing recurrences of the clinical status" in brief also "continuing care" [6]. These definitions resemble that used by Breen, when reporting his study, in 1976: "... treatment, either scheduled or elective, which occurred after optimum recorded benefit was reached, provided there was no evidence of relapse." [15]. But on the other hand, according to a recent British study of chiropractors, osteopaths and physiotherapists, at least 10% of each profession reported that they sometimes continue to treat patients with low back pain who show almost no improvement [14]. Therefore, it appears that maintenance care is also offered to patients who have *not *improved. Boline and Sawyer [10] report on "regular chiropractic care", which we interpreted as meaning maintenance care.

Other authors, who studied this subject, did not define maintenance care at all [9,13,16,17] and in one study such non-definition was stated to be purposeful, in order to prevent bias of the investigation process [11].

### Indications for maintenance care

There was only sparse information in the literature on the indications for maintenance care and there was no information on specific indications for particular conditions.

The chiropractors in a North-American study by Rupert [11] generally agreed that the purposes of maintenance care were to minimize recurrence or exacerbation, maintain or optimize state of health, prevent conditions from developing, provide palliative care for "incurable" conditions, and determine and treat subluxations (all statements with over 80% agreement). Fifty-six percent meant that the purpose of maintenance care was to prevent subluxations, and this was confirmed in a similar study of Australian chiropractors [13]. Most of these statements reflect secondary or tertiary preventive approach. Nevertheless, some could also be interpreted as referring to a primary preventive approach.

Interestingly, the concept of "prevention and health promotion" was used in a North American study of maintenance care in relation to chiropractic patients aged at least 65 [12]. Boline and Sawyer explored similar concepts, also in a study from North America, in which they investigated attitudes among chiropractors in relation to counselling of patients on a healthy lifestyle, i.e. including elements of primary prevention, as well as regular chiropractic care. [10]. This shows that they mean that chiropractors should participate in health promotion and prevention of disease, outside the realm of the purely musculoskeletal.

According to a prospective multicenter study of 115/205 Norwegian chiropractors and 832 patients with persistent low back pain, patients, who after one year reported definite improvement, were treated only a few times and those with poorer outcome had a larger number of consultations over a period of one year. This might indicate that tertiary prevention is offered to and accepted by some of those patients with persistent low back pain, who fail to recover, but that secondary prevention in patients with more satisfactory outcome is less common [17]. This information is the only indication of how maintenance care is actually employed in clinical practice.

### The nature of the use of maintenance care

According to the Mercy Guidelines [7], spinal adjustments are central in maintenance care. However, the research literature is vague on the contents of a maintenance care consultation and the frequency of treatments required for different types of patients.

Rupert [11] asked his North-American study subjects to describe the therapeutic components of maintenance care and concluded that they were adjustments/spinal manipulation, exercises, proper eating habits, patient education, and vitamin supplementation. This finding was confirmed in a similar a study of Australian chiropractors [13] and in another study of Rupert et al in North American patients, aged 65 or older [12].

Wenban described the outcome in relation to the various outcomes measures used when treating one female patient with a complaint of uncomplicated chronic low back pain [18]. His indications for treatment were "tenderness of the patient's vertebral spinous processes, S2 spinous process, and the superior aspect of the posterior superior iliac spines". According to the author, this indicated a "subluxation", and providing that there were no other "more serious indicators" (not further specified), treatment was provided. The treatment consisted of adjustments only, using a combination of diversified and sacro-occipital techniques (a reference is provided to two textbooks].

Wenban also described the frequency of visits, after a 12 weeks intensive care period, as 2 times per week for 6 weeks, 1 time per week for 2 weeks, and 1 time per 2 weeks for the reminder of the study period of 5.5 months. In addition, Jamison offers some information on this subject. According to the participants in one of her studies: "Maintenance adjustments should be offered on a basis of once a month to once every three to four months" [4]. According to one of Rupert's studies, North American patients who agree to receive maintenance care, average 14 visits per year [11], and in another study, elderly maintenance care patients (65+ years of age) average 17 visits per year [12]. This amounts to a little more than one visit per month.

There were no studies of different types of strategies in relation to different types of patients or conditions.

### Beliefs among chiropractors and acceptance among patients

Boline and Sawyer in a 1987-survery of North American chiropractors noted that 98% of their participants believed that "regular chiropractic care would be important for the 'average' person" [10]. Jamison found that 93% of Australian chiropractors considered that at least some patients would require maintenance care (and that 41% thought that all patients would) [4]. In another survey, she found that 92% believed that spinal adjustments promote health in asymptomatic patients [5].

However, the last two studies dealt primarily with the concept of spinal adjustments and the prevention of endocrine, visceral and autonomic dysfunction, which may have incited chiropractors of specific opinions to participate and, indeed, the very low response rates (22% and 20%, respectively) indicate that this may have been the case.

These beliefs are underpinned by the finding in the study by Rupert [11], in which 40% of the chiropractic respondents believe that there is adequate research to support the concept of maintenance care. The Australian respondents were less naïve, with only 22% supporting this statement [13].

We found no information on the patients' perspective of maintenance care. However, in one study it was stated that 79% of patients are recommended for maintenance care and that 34% of those "elect to receive these services" [11]. It is not clear, whether these estimates are based on an objective count of patient files or on the participating chiropractors' opinions. However, the figure of 34% does not appear unreasonable, given the personal experience of many chiropractors that only some patients are willing and able to continue treatment past the acute event.

Even among patients with persistent low back pain, maintenance care might not be attractive. Of the 832 participants in a large Norwegian multi-centre prospective outcome study, all with persistent low back pain at base line, only 14% were reported by their chiropractors to have received some type of maintenance care during the subsequent year [17].

In summary, it is possible that most chiropractors believe in maintenance care but data from unbiased samples are missing. The opinion of patients is unknown, although it might be less positive, as a relatively low percentage of patients seem to accept to continue treatment past the initial treatment program.

### Prevalence of use and factors associated with its use

Although the concept of maintenance care seems to be firmly ensconced in the chiropractic profession, the frequency of its use has not been clearly described.

Breen reported in 1976 that 36% of 2987 case files belonged to patients who received maintenance care [15]. These patients were obtained from a sample belonging to 24 British chiropractors, i.e. 35% of the practitioners at that time. They had been selected to give a proportional representation of practitioners in the British hospital regions, and a 20% randomly selected sample of up to 1000 case files was taken from each practice. There were proportionally fewer files from newly established practices. Most of these chiropractors were educated in North America, practising at a time when there were relatively few manipulating practitioners but also at a time when chiropractic was relatively unknown. It was noted that most patients consulted for "rheumatic conditions and in particular low back pain" whereas non-musculoskeletal problems were very rare. This appears to be the first investigative study on this subject, and it probably provides a fair picture of the use of maintenance care in the UK at that time.

Others have reported the proportion of patients who receive maintenance care in the literature. Webb and Leboeuf in 1987 found that 44% of newly graduated Australian chiropractors estimated that at least 34% of their patients were on maintenance care [9].

A similar estimate was obtained in another Australian study published two years later [16]. In both these two studies, only 6% of the respondents reported that more than 2/3 of their patients received maintenance care. However, although the response rates were better in these, two studies (65% and 82%, respectively); their estimates were based on the practitioners' opinion rather than exact counts of patient files.

Obviously, the use of maintenance care will affect the clinic income. According to Rupert's participants, 23% of practices' incomes was generated from maintenance care [11]. That the use of maintenance care can affect the patient turnover was shown in the Leboeuf et al study from Australia [16].

We were unable to find any information on which factors play a role, when a chiropractor decides to offer a maintenance care program to a patient, and no information seems to be available on what considerations patients take into account when deciding to accept such a program.

### Efficacy and cost-effectiveness for various types of conditions

It remains also to study the efficacy and cost-effectiveness of maintenance care for various types of conditions.

To our knowledge, it has only been attempted to test the efficacy of maintenance care in one well-designed pilot study, in which 29 patients with chronic low back pain were randomly allocated to either a non-maintenance care group or a maintenance care group (for a 9-month treatment after one initial month of treatment). At follow-up, there was no difference in pain but the group that received maintenance care had lower disability scores than the control group [19]. A full-scale trial is presently underway on patients with neck pain (personal communication – M. Descarreaux).

## Discussion

This literature review reveals that more than 30 years of ad hoc research into maintenance care does not provide much information. In fact, the relative paucity of studies, the obvious biases in many of these, the lack of exhaustive information, and the diversity of findings made it impossible to answer any of our questions.

It is not clear whether chiropractors use maintenance care mainly as a secondary or tertiary preventive measure, and if so how frequently and for which conditions. It is possible that there are considerable differences between countries and perhaps during certain periods. The Australian literature in the 1990s dealt with some fundamental concepts, such as "the healthy spine – freedom from disease" concept, which the UK study 20 years earlier did not concern itself with. In between, Mitchell in 1980 [6] transmitted a warning on over-servicing, which is the negative side of maintenance care if used unwisely. None of the studies looked at this very difficult balance.

Several studies indicated that almost all chiropractors believe in the value of maintenance care, but newer studies on unbiased study samples are needed to confirm these findings. In addition, it is possible that differences exist between different groups of chiropractors. Would chiropractors who graduated from a university-based chiropractic institution have the same beliefs as those coming from chiropractic colleges of a more traditional type? Further, the density of chiropractors in a region might have an effect on this aspect.

Patients' opinions and expectations of maintenance care and their satisfaction with maintenance care appear not to have been studied. This is, nevertheless, an important subject. Unless expectations and results match, patients are likely to become unsatisfied.

The treatment program, including contents and time schedule, requires further study. Only one report deals explicitly with the timing of treatments. Wenban [18] describes a pre hoc determined and rigid plan for a large number of treatment visits in a young patient with "uncomplicated" low back pain for more than three months. We know, from personal experience that also other, more flexible, treatment plans are used, but how common are these respective approaches?

Despite the bulk of the literature being of poor quality, there seems to be light at the end of the tunnel. It has been shown that it is feasible to conduct a randomized controlled clinical trial of maintenance care, and a full-scale study is presently underway. However, several aspects other than efficacy need to be investigated. For example, it would be relevant to test the cost-effectiveness for patients who choose to be treated regularly vs. those who receive treatment only when symptoms arise. This should be investigated for different types of conditions and using different treatment programs. In other words, the aim should be to be able to say, not only, if maintenance care is a clinically valid treatment approach but, also, for whom it should be used, how and when.

However, before testing the clinical validity of maintenance care, it would be necessary to find out what it is, how it is practised and on which indications, to make sure that such a trial does reflect the situation in real life.

## Conclusion

• There is no evidence-based definition of maintenance care and the indications for and nature of its use remains to be clearly stated.

• It is likely that many chiropractors believe in the usefulness of maintenance care but it seems to be less well accepted by their patients.

• The prevalence with which maintenance care is used has not been established.

• Efficacy and cost-effectiveness of maintenance care for various types of conditions are unknown.

Therefore, the recommendations given by Aker and Martel [8] more than a decade ago are still relevant, namely that "before a large-scale, multicentered clinical trial can be pursued, a series of preliminary studies need to be conducted to delineate the parameters of care to be used in the clinical trial, the outcome measures to be used, and the feasibility of conducting such a complicated and resource-intensive study."

## Competing interests

The authors declare that they have no competing interests.

## Authors' contributions

CY did the literature search. Both authors read the articles and abstracted the data. CY made the first draft of the manuscript. LH revised it critically for intellectual content. Both authors read and approved the final manuscript.

## Supplementary Material

Additional file 1 Table 1. Description of 11 surveys in a review on the use of maintenance care among chiropractors. The table provides an overview of the reviewed articles, including author, year of publication, year and country of study, population, response rate, sampling method, data collection and objectives as they relate to the present review. Click here for file
