# Supplementary material for: Maintenance care in chiropractic – what do we know?
Source: Chiropr Osteopat. 2008 May 8;16:3. doi: 10.1186/1746-1340-16-3 (PMC2396648; doi:10.1186/1746-1340-16-3)
Supplement: Additional file 1 — Table 1. Description of 11 surveys in a review on the use of maintenance care among chiropractors. The table provides an overview of the reviewed articles, including author, year of publication, year and country of study, population, response rate, sampling method, data collection and objectives as they relate to the present review. [file 1746-1340-16-3-S1.doc]

**Table 1. Description of 11 surveys in a review on the use of maintenance care among chiropractors.**

| Author. Year of publication. | Year of data collection | Country  of study | Target  population | Number of participants  (response rate) | Sampling  method | Data collection | Those objectives of the study, that relate to the objectives of this review** |
| --- | --- | --- | --- | --- | --- | --- | --- |
| Breen  1976 [15] | 1973-4 | UK | Chiropractors  in UK | 24 DCs*  (35%)  2056 case files | DCs*: Proportional representation in the hospital regions.  Case files:  20% randomly selected case files.  Proportionally fewer cases in newly established clinics. | Inspection of case files | 1a  3a |
| Webb &  Leboeuf  1987 [9] | 1985 | Australia | Newly graduated  chiropractors in Australia | 131DCs*  (65.5%) | 150 Phillip Institute graduates and 50 newly graduated non-Phillip Institute graduates | Questionnaire | 3a |
| Leboeuf, Morrow & Payne  1989 [16] | 1986-7 | Australia | Chiropractors practicing in either areas densely or sparsely populated by chiropractors | Baseline: 99 DCs*  (82%)  Follow-up:  74 DCs  (61%) | Selected on the basis of practice location based on density of chiropractors IN Victoria and South Australia | Questionnaires | 3a  3b |
| Boline &  Sawyer  1990 [10] | 1987 | USA | Chiropractors licensed to practice in the state of Iowa. | 374 DCs*  (51%) | Probably the whole study population in one US state | Questionnaire | 1a  2a |
| Jamison  1991 [4] | ? | Australia | The whole study population in Australia | 285 DCs*  (22%) | Chiropractors members of the two recognised chiropractic associations | Questionnaire | 1c  2a |
| Jamison  1991 [5] | ? | Australia | The whole study population in Australia | 339 DCs*  (20%) | Chiropractors listed by the Australian Chiropractors’ Association in 1990 | Questionnaire | 2a |
| Rupert  2000 [11] | ? | USA | Chiropractors practising in USA | 658 DCs*  (44%) | Random selection, weighted by size of the state ensuring participants from each state of US and rural areas and large cities. | Questionnaire | 1b  1c  2a  2b  3b |
| Rupert, Manello & Sandfuhr  2000 [12] | 1995 | USA | Chiropractors from 6 study sites | 73 DCs*  (?%)  311 patients  (?) | Chiropractors selected for using maintenance care and elderly patients receiving maintenance care | Questionnaires | 1b |
| Jamison & Rupert  2001 [13] | 1995 and probably 1999 | USA  Australia | US data the same as in Rupert 2000  Chiropractors listed by the Chiropractors’ Association of Australia in 1999 | US data see Rupert 2000  Australia:  138 DCs*  (35%) | See Rupert 2000 for US study.  Randomly selected members of the Chiropractors’ Association of Australia | Questionnaire. | 1b  1c |
| Leboeuf-Yde, Grønstvedt, Borge et al 2004 [17] | 2000 | Norway | All registered chiropractors in Norway invited 10 consecutive patients each. | 1-yr follow-up (data from 115/205 (56**%**) DCs* on  832/875 patients  (95**%)** | All Norwegian chiropractors.  Consecutive patients with LBP > 30 days in the past year and >2 wks at time of consultation | Questionnaire | 1b  3a |
| Pincus, Vogel, Breen, Foster & Underwood  2006 [14] | ? | UK | Chiropractors, (osteopaths and physical therapists) | Questionnaire:  122 DCs* (61%)  Interview:  11 DCs* (continued treatment)  2-4 DCs* (did not continue treatment) | Questionnaire:  Randomly selected members of chiropractic, osteopathic and physiotherapy professions  Interview: Those who continued to treat patients without improvement and some who did not. | Questionnaire and interviews | 1a |

* DC: Doctor of Chiropractic

** Objectives: 1a. Definition of maintenance care

1b. Indications for maintenance care

1c. The nature of the use of maintenance care

2a. The degree of belief in maintenance care among chiropractors

2b. The degree of acceptance of maintenance care among patients

3a. Establishing the prevalence of the use of maintenance care

3b. Factors associated with the use of maintenance care

4. Efficacy and/or cost-effectiveness of maintenance care
